# Does Temporary Baiting Affect White‐Tailed Deer Space Use and Movement? New Insights Leveraging Subhourly Location Data

**DOI:** 10.1002/ece3.72936

**Published:** 2026-01-19

**Authors:** Dylan G. Stewart, Jared T. Beaver, M. Lucas Cooksey, Chad Grantham, Brian L. Pierce, Roel R. Lopez, Stephen L. Webb

**Affiliations:** ^1^ Department of Rangeland, Wildlife, and Fisheries Management Texas A&M University College Station Texas USA; ^2^ Department of Animal and Range Sciences Montana State University Bozeman Montana USA; ^3^ Texas A&M Natural Resources Institute San Antonio Texas USA; ^4^ United States Department of Agriculture – Natural Resources Conservation Service Waxahachie Texas USA; ^5^ Texas A&M Natural Resources Institute College Station Texas USA

**Keywords:** bait, camera survey, *Odocoileus virginianus*, space use, supplemental feeding, Texas

## Abstract

Temporary baiting is often used to increase detection rates during camera surveys, particularly for white‐tailed deer ( *Odocoileus virginianus* ). However, the effects of bait on deer space use and movement have not been examined using location data sampled at intervals matching typical bait‐site visits. We defined three bait periods (pre‐bait, bait, post‐bait) to evaluate the effects of presence and removal of bait (i.e., shelled corn) on deer space use and movement relative to control deer not exposed to bait. We captured and fitted 61 deer (32 F, 29 M) in central Texas with GPS collars, that attempted a GPS location every 15 min from July 22 to September 2, 2012–2013. Of these (3 recaptures), 41 (20 F, 21 M) and 23 (15 F, 8 M) collars were assigned to the impact and control groups, respectively. We used generalized linear mixed‐effects models to evaluate the effect of bait on daily estimates of range size (ha), range overlap (proportion, 0–1), distance of activity centers to the nearest bait site (m), and distance traveled (m/15‐min) by deer. Deer exposed to bait expanded ranges and moved more during the active period (crepuscular, night) but moved less during the inactive period (day) relative to the pre‐bait period, indicating complex, time‐dependent responses. Deer exposed to bait did not shift their activity centers toward bait sites when bait was present. After bait removal, deer continued foraging within previously established ranges rather than abandoning the area. These results suggest that temporary baiting is unlikely to attract deer from outside their normal ranges, and bait removal is unlikely to cause deer to leave established areas, preserving the closure assumption. Our findings have implications for wildlife managers concerned about post‐bait movements and improve understanding of time‐dependent behavioral responses to supplemental food resources.

## Graphical abstract

We evaluated the impact of presence and removal of bait (i.e., shelled corn) on range size (ha), range overlap (proportion, 0–1), distance of activity centers to bait sites (m), and distance traveled (m/15‐min) by deer in central Texas from 2012 to 2013. Deer exposed to bait did not shift their activity centers toward bait sites when bait was present, and, after bait removal, they did not abandon the area in search of new resources but continued to forage within previously established ranges. These results suggest that temporary baiting is unlikely to attract deer from outside their normal ranges, and bait removal is unlikely to cause deer to leave established areas, preserving the closure assumption.

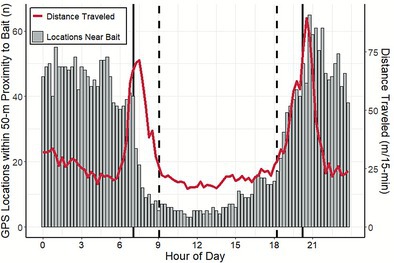

## 1 Introduction

Baiting is the intentional use of artificial or natural feed to alter the behavior of wild species by attracting wildlife to a localized area (Inslerman et al. 2006; Sorensen et al. 2014). Common objectives for baiting wildlife include (1) increasing hunting success (Dunkley and Cattet 2003; Powell and Proulx 2003; Kilpatrick et al. 2010), (2) enhancing viewing opportunities (Knight 2009), (3) capturing wildlife for research purposes (e.g., for reintroduction, population augmentation, disease testing, and monitoring; Naugle et al. 1995; Webb, Lewis, et al. 2008; Lula et al. 2020), (4) trapping and controlling overabundant native or invasive species (Baker et al. 2005; Kilpatrick et al. 2010; Bowman 2011; Snow et al. 2017; Taggart et al. 2023), and (5) increasing encounter probability for camera surveys (Du Preez et al. 2014; Johnson et al. 2021; Navarre 2021). The use of supplemental feeding, and by extension baiting, can be a contentious topic with some supporting it while others oppose it (Dunkley and Cattet 2003; Brown and Cooper 2006; Hansen 2011). One major concern is that baiting can alter the spatial distribution of wildlife, thereby artificially inflating population density, disrupting daily or seasonal movements, or attracting wildlife to private land or unhunted sanctuaries (Dunkley and Cattet 2003). Long‐range movements (up to 5.4 km) by deer to feed on bait have been documented (Van Brackle et al. 1995); however, others have found that baiting had minimal effects on home range size (Kilpatrick and Stober 2002; Campbell et al. 2006; Cooper et al. 2006; Rustand 2010), suggesting these movements are atypical or region‐specific. Indeed, occurrence and frequency of these cross‐boundary movements likely depend on the size of the property, quality of the habitat, and placement of bait relative to established home ranges. Moreover, temporary baiting (i.e., few days to weeks) likely has a different effect on deer than prolonged baiting or supplemental feeding (i.e., months).

Bait dispensed within home ranges of white‐tailed deer ( *Odocoileus virginianus* ) may influence feeding behavior and activity patterns, particularly among juveniles (Saldo et al. 2024), leading to increased detection rates during camera surveys (Macaulay et al. 2019; Johnson et al. 2021; Navarre 2021). Common deer abundance estimators, such as the Jacobson method (Jacobson et al. 1997), integrate use of bait into the survey design to maximize detections, thereby shortening the sampling period (Macaulay et al. 2019). For example, Johnson et al. (2021) reported that cameras collected 40–50 times more images of antlered males at bait sites compared to unbaited sites, resulting in a greater encounter probability. Similarly, Navarre (2021) reported that deer detections increased 148‐fold at bait sites compared to unbaited sites.

Most studies that have investigated the effects of baiting on deer space use have found little to no impact on home range sizes (Kilpatrick and Stober 2002; Campbell et al. 2006; Cooper et al. 2006; Webb, Hewitt, et al. 2008; Rustand 2010). Instead, deer often shift centers of activity (i.e., core area; Campbell et al. 2006) closer to bait sites (Williams and DeNicola 2000; Kilpatrick and Stober 2002; Campbell et al. 2006; Rustand 2010), or core areas may fragment to encompass multiple bait sites (Williams and DeNicola 2000). While some studies documented a reduction in core area sizes in response to baiting (Williams and DeNicola 2000; Cooper et al. 2006), others have reported no significant change (Kilpatrick and Stober 2002; Campbell et al. 2006). Additionally, Garver (2011) tested the effect of supplemental feed on deer movement rates and found that movement rates were greater at night for deer provided supplemental feed.

Forage availability and quality across the landscape, as well as the duration of bait or crop exposure, may influence the effect of bait on deer space use and movement. Animals are likely to reduce use of supplemental feed during periods of green‐up or mast production (Roden‐Reynolds et al. 2022), which may also limit resource dependency and search behavior following bait removal. Likewise, extended exposure to bait or crops may increase dependency and exacerbate the effects of bait presence and removal on space use and movements. Most prior studies incorporated baiting periods ranging from 21 to 99 days (Campbell et al. 2006, 2012; Williams and DeNicola 2000; Vercauteren and Hygnstrom 1998; Kilpatrick and Stober 2002), typically reflecting long‐term baiting or supplemental feeding, which may elicit different responses compared to short‐term baiting. An additional consideration is the frequency of location data collection. Previous studies using VHF telemetry or GPS technology analyzed location data collected at 30‐min to > 4‐h intervals to quantify the effects of baiting on deer space use and movements (Williams and DeNicola 2000; Campbell et al. 2006; Cooper et al. 2006; Webb, Hewitt, et al. 2008; Rustand 2010; Garver 2011). However, the average deer visit duration at baited camera sites can be less than 15 min (Ozoga and Verme 1982; Kozicky 1997; Newbolt et al. 2017). As such, these studies may have captured feeding events disproportionately to their occurrence on the landscape, which could result in an under‐ or overestimate of the effect of baiting.

We are unaware of a study that has examined the effects of temporary bait presence and subsequent removal on deer space use and movements using location data sampled at a frequency matching visit durations of typical visits at bait sites. Therefore, we conducted an experiment using location data collected at 15‐min intervals to quantify deer space use and movement before temporary bait was placed, while temporary bait was present, and after temporary bait was removed. We predicted that (1) deer would move closer to bait sites and increase their movement (Williams and DeNicola 2000; Kilpatrick and Stober 2002; Campbell et al. 2006; Rustand 2010; Garver 2011) while range size and range fidelity would remain largely unchanged during the baiting period compared to pre‐bait conditions (Kilpatrick and Stober 2002; Campbell et al. 2006; Cooper et al. 2006; Webb, Hewitt, et al. 2008; Rustand 2010); and (2) following bait removal, deer would increase range size, decrease range fidelity, move farther from bait sites, and continue elevated movements relative to the baiting period (Vercauteren and Hygnstrom 1998; Campbell et al. 2012), indicative of search behavior.

## 2 Materials and Methods

### 2.1 Study Area

**FIGURE 1:**
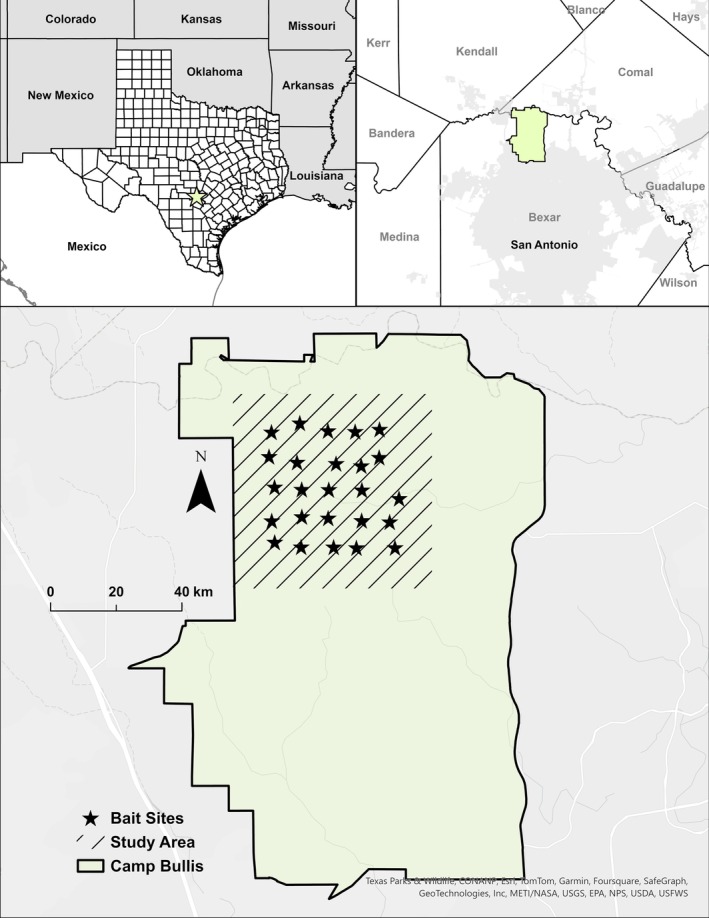
Study area (diagonal lines) and bait site locations (stars) within Joint Base San Antonio–Camp Bullis (solid black outline), San Antonio, Bexar County, Texas, USA, during 2012–2013. The map was created by D. G. Stewart using ArcGIS Pro v3.5.3 (Esri, Redlands, CA, USA).

We conducted our research at Joint Base San Antionio‐Camp Bullis (JBSA‐CB), a 11,286‐ha U.S. Army training facility located within the Edwards Plateau, Blackland Prairies, and South Texas Plains ecoregions north of San Antonio, Bexar County, Texas, USA (Gould 1962; Beaver et al. 2022; Stewart, Beaver, et al. 2025; Figure 1). We provide a brief study area description and overview of the animal capture and handling procedures; full details are available in Beaver et al. (2022) and Stewart, Beaver, et al. (2025). The two dominant cover types on Camp Bullis were scrub evergreen and upland deciduous forest (Van Auken et al. 1979, 1981). Scrub evergreen forests were dominated by Ashe juniper ( *Juniperus ashei* ), plateau live oak ( *Quercus virginiana* ), and Texas persimmon ( *Diospyros texana* ) interspersed across hilltops and southwestern aspects of hill slopes (Van Auken et al. 1981). Upland deciduous forests were characterized by Spanish oak ( *Quercus texana* ), Lacey oak ( *Quercus glaucoides* ), Ashe juniper, and Texas persimmon dispersed along the northeastern aspect of hill slopes (Van Auken et al. 1981).

Elevation ranged from 320 to 440 m above sea level (USGS 2023). The soil was dominated by Brackett gravelly loam (39%), Krum clay (15%), and Eckrant cobbly clay (25%), with a range of 1%–60% slope (NRCS 2023). The mean annual temperature was 20°C for the study site, with monthly averages ranging from 11°C in January to 30°C in July. Annual rainfall ranged from 36 to 89 cm. Plant growth peaked from March to June and from September to October, correlating with months of maximum precipitation (Taylor et al. 1966).

### 2.2 Capture and Handling

We captured 123 individual deer (137 capture events) using drop nets released from the ground or a net gun deployed from a helicopter. Captures took place in August, September, and November 2011, February, March, June, and July 2012, and February and July 2013. Capture efforts were constrained to the northwestern section of the installation to ensure safety of military personnel and reduce human‐wildlife interactions. For ground captures, we used 20 × 20‐m nylon drop nets suspended 2–3 m above the ground and baited with shelled corn; drop nets were manually released by a researcher positioned nearby (Lopez et al. 1998). All deer were released at the capture site. Drop net capture sites were chosen to provide uniform coverage across the study site while maximizing capture opportunity. For aerial captures, we used a single helicopter or a set of two helicopters (Robinson 22, Robinson Helicopter Company, Torrance, CA, USA). For single helicopter captures, we located, pursued for a maximum of 10 min, captured via a 4‐barreled netgun, and transported (< 0.5–3.1 km) deer to a processing station (DeYoung 1988; Webb, Lewis, et al. 2008). Similarly, we used a team of two helicopters, working in consort, whereby one helicopter captured and prepared deer for transportation while the second helicopter located, herded, and transported (< 0.5 km) deer to research vehicles that followed the helicopters and processed deer on site (DeYoung 1988). All deer were released from the central processing site or a mobile processing station.

All deer were blindfolded upon capture or arrival at the processing site, marked with a unique ear tag, aged using tooth replacement and wear methods (Severinghaus 1949), and sexed (male, female). Each deer was fitted with a GPS neck collar (Model G2C 191; Sirtrack, Havelock North, New Zealand), programmed to remotely release approximately 90 days following capture. All animal procedures were approved by the Texas A&M University Institutional Animal Care and Use Committee (AUP# 2011‐154).

### 2.3 Data Preparation

We programmed GPS collars to record locations every 15‐min (96 fixes/day). The longitude, latitude, timestamp (UTC + 0), horizontal dilution of precision, and number of satellites associated with each fix were stored on board each collar. Once collars were recovered, we downloaded location data, converted timestamps to local mean time (CDT [UTC‐5], CST [UTC‐6]) and calculated Euclidean distance (m) between successive locations. Additionally, we calculated sunrise and sunset times for each day using the *sunrise* and *sunset* functions from the bioRad package (v. 0.8.1; Dokter et al. 2019) in R statistical software (v. 4.3.0; R Core Team 2023) and then categorized locations as occurring during the crepuscular periods (30 min before to 2 h after sunrise [morning crepuscular period] and 2 h before to 30 min after sunset [evening crepuscular period]), daylight hours (2 h after sunrise to 2 h before sunset), and night hours (30 min after sunset to 30 min before sunrise; Stewart et al. 2022).

We followed methods outlined by Stewart, Beaver, et al. (2025) to confirm or estimate the date each collar detached or a mortality event occurred (i.e., the end date), and to identify and censor erroneous GPS locations, while minimizing data loss. First, we identified when collar detachment or mortality events occurred using the *flag_defunct_clusters* function from the amt package (v. 0.2.2.0; Signer et al. 2019). Next, we estimated the distance between each GPS location and the median longitude and latitude using the *outlie* function from the CTMM package (v. 1.2.0; Calabrese et al. 2016; Fleming and Calabrese 2022) and censored fixes > 32 km; the median longitude and latitude were estimated for each deer to account for individual differences in movement. Last, we censored locations that were ≥ 2 km (usually outside JBSA‐CB) from the previous location and the location following an erroneous location to avoid artificially inflating movement metrics. This approach retained the maximum amount of usable data while effectively removing outliers that could distort daily movement estimates.

### 2.4 Camera Survey

We performed a baited camera survey from August 6 through August 17, 2012 and from August 5 through August 19, 2013, which aligned with a period during which deer surveys are commonly conducted in the southeastern United States (Koerth et al. 1997; Johnson et al. 2021). We established a systematic array of 25 camera sites across 1,425 ha within the JBSA‐CB study area boundary (Figure 1), resulting in 1 camera per 57 ha (Jacobson et al. 1997). We generated a 755 × 755 m grid of 25 cells (5 × 5) using ArcGIS Pro (ESRI 2023) then camera sites were centered in each grid cell (Beaver 2017). Established locations deviated slightly from center based on topography, likelihood of visitation by deer, and ease of access (Beaver 2017). At approximately 0700 (CDT) on August 6, 2012 and August 5, 2013, we placed 12.5 kg of shelled corn in a pile at each camera site and replenished the bait every 2 days throughout the survey period (Beaver 2017). We removed bait at roughly the same time on August 17, 2012 and August 19, 2013.

We implemented a BACI design by creating three bait periods (pre‐bait [before], bait [during], post‐bait [after]), which were compared to deer not exposed to bait (control). The pre‐bait and post‐bait periods were 14 days in length, and the bait period was 11 days in 2012 and 14 days in 2013. Since bait was dispensed in the morning hours on the first day of the bait period and bait was removed in the morning hours of the first day of the post‐bait period, we created 24‐h periods, which we termed Days that extended from 2 h after sunrise (end of the morning crepuscular period) to the same time the following day. The baiting period lasted from the end of the morning crepuscular period on 6 August to the same time on August 17, 2012 and followed the same start and end times on 5 August and 19 August in 2013. Similarly, the pre‐bait period spanned from 22 July to 5 August, and the post‐bait period from 19 August to 2 September, with both starting and ending 2 h after sunrise. We removed 5, 17, and 18 August 2012 from analyses to create two sampling periods spanning the same range of dates.

### 2.5 Data Analysis

We selected deer for inclusion in the impact (exposed to bait) and control (not exposed) groups based on three criteria. First, we included only adult deer (> 18 months old at capture) that were collared and actively collecting data for the entire 42‐day study period. Second, we restricted our analysis to individuals that remained within the boundaries of JBSA‐CB throughout the study. Although the facility is enclosed by a ~2.4‐m‐high perimeter fence, flooding events occasionally created breaches that allowed deer to cross the boundary. These compromised sections were subsequently repaired by military personnel, which may have limited access to bait sites (Stewart, Beaver, et al. 2025). Last, we classified deer as part of the impact group if they had access to at least one baited camera site. To determine access, we created 99% utilization distributions using Brownian bridge movement models (BBMMs) for each individual using GPS data collected during the 42‐day study; we used the *kernelbb* and *getverticeshr* functions from the adehabitatHR package to estimate BBMMs (v. 0.4.22; Calenge and Fortmann‐Roe 2023). We derived the maximum likelihood estimation using the *likr* function (Calenge and Fortmann‐Roe 2023) where the second smoothing parameter was set to 20 m, a conservative estimate when relocation error is unknown (Stewart, Mendes, et al. 2025). For each BBMM, we set the first smoothing parameter to the maximization of the log‐likelihood derived from the *likr* function, set the second smoothing parameter to 20 m, and centered the utilization distribution on a 500‐m spatial grid (Calenge and Fortmann‐Roe 2023).

**FIGURE A1:**
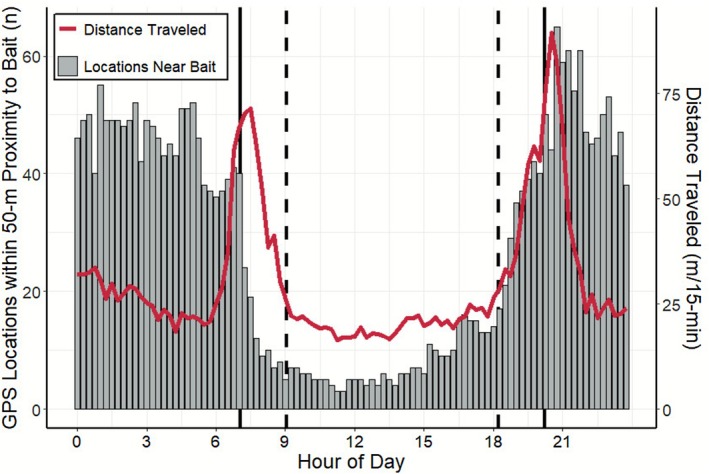
Relationship between time of day and the number of GPS locations within a 50‐m proximity to bait sites (gray bars–y‐axis #1) or the median distance traveled (m/15 min; red line–y‐axis #2) for white‐tailed deer ( *Odocoileus virginianus* ) during baited surveys at Joint Base San Antonio–Camp Bullis, Texas, USA, in 2012–2013.

We categorized location data into two temporal activity periods based on established movement patterns and proximity to feeders. The active period encompassed evening crepuscular, night, and morning crepuscular hours, times when deer typically exhibit increased movement (Webb et al. 2010; Stewart et al. 2022) and are most frequently located near feeders (Figure A1). We applied a distance threshold of 50 m from feeders to visualize activity and proximity patterns, which supported grouping crepuscular and nocturnal hours due to their similar spatial behaviors. The active period was categorized from approximately 1812 to 0902 CDT, while the inactive period spanned the intervening hours. Together, the active and inactive periods constituted a complete 24‐h cycle within the previously defined Day period (Figure A1).

We used daily estimates of space use and movement to assess the effect of bait on spatial distribution patterns of deer (Long et al. 2018). We created 99% utilization distributions using BBMMs for each individual, day, activity period combination that included 24 or more locations, which was necessary to obtain a stable estimate (Campbell et al. 2006). We fit BBMMs using the same approach and parameters as described for determining access to bait sites. We used the *st_area* and *st_intersection* functions from the sf (v. 1.0‐19) package (Pebesma 2018; Pebesma and Bivand 2023) to estimate daily range size (ha/day) and the proportion of overlap (0–1) between daily ranges by activity period. We quantified the distance of deer activity centers to the nearest bait site (m) by calculating the Euclidean distance between the geometric center of each range isopleth and the nearest bait site using the *st_centroid* and *st_distance* functions in the sf package (Vercauteren and Hygnstrom 1998; Campbell et al. 2006). We evaluated the effect of bait on distance traveled (m/day) by deer by averaging sequential step lengths (15‐min intervals) for individuals during each activity period daily. We omitted step lengths (< 0.4% of location data) where the time between sequential locations was ≤ 10‐min or ≥ 20‐min (Stewart et al. 2022). We selected range size, range overlap, distance of deer activity centers to the nearest bait sites, and distance traveled for evaluation because these metrics represent fundamental and widely applied indices of space use and movement in ecological research, facilitate comparisons across studies, and are influenced by the spatial configuration and availability of resources (Millspaugh and Marzluff 2001; Campbell et al. 2006; Webb et al. 2010; Bunnefeld et al. 2011; Hewitt 2011; Stewart, Mendes, et al. 2025).

We developed a covariate for woody vegetation to account for its influence on space use and movement. We used Google Earth Engine (Gorelick et al. 2017) to download 30‐m spatial resolution Rangeland Analysis Platform (Allred et al. 2021) shrub and tree raster layers for 2012 and 2013, which we then combined for each year to produce single woody cover layers. We evaluated the influence of woody vegetation as opposed to herbaceous cover because deer are dependent on woody cover to thermoregulate in arid systems (Dykes 2022), particularly during the summer months, and cover is an important component of habitat (Chance et al. 2020). For each individual, we calculated mean daily percent woody cover by averaging raster values within their daily activity‐specific range isopleths.

We used generalized linear mixed‐effects models, implemented using the *glmmTMB* function from the glmmTMB package (v. 1.1.10; Brooks et al. 2017), to evaluate the effects of bait presence and removal, activity period, treatment, and sex on daily range size (ha/day), proportion of daily range overlap (0–1), distance of deer activity centers to the nearest bait site (m/day), and mean distance traveled (m/15‐min/day). We used the *simulateResiduals* and *testDispersion* functions from the DHARMa package (v. 0.4.7; Hartig 2024) to generate QQ plots, residual plots, and dispersion plots to assess model diagnostics and determine the most appropriate distribution and link function for the data. We developed five models using a BACI design, two of which (range overlap, distance traveled) included five explanatory variables: a categorical variable for “bait period” with three levels (pre‐bait, bait, post‐bait), a categorical variable for “sex” with two levels (female, male), a categorical variable for “treatment” with two levels (impact, control), a categorical variable for “activity period” with two levels (active, inactive), and a continuous covariate representing woody vegetation cover. We developed separate models for range size during the active and inactive periods, using the same model structure described above, because range size estimates may be different between activity periods, stemming from a greater volume of location data during the active period. We parameterized a model for distance of deer activity centers to the nearest bait sites using the same model structure described above but excluded the treatment effect because deer in the control group were known a priori to be farther from bait sites. We included a woody vegetation cover covariate to account for its influence on space use and movement. Prior to analysis, we centered and scaled the woody cover covariate by subtracting the population mean and dividing by the standard deviation. We included random effects for unique collar events to account for unbalanced data and correlation within each individual deer, and for year to account for interannual variation (Muff et al. 2020).

Range size and distance to bait site data were strongly right‐skewed; therefore, to meet assumptions of normality and homoscedasticity, we normalized range size values using a natural‐log transformation and distance to bait site values using an ordered quantile transformation, implemented using the *orderNorm* function from the bestNormalize package (Peterson and Cavanaugh 2020; Peterson 2021). Ordered quantile normalization was used because standard parametric transformations (e.g., log transformation) did not adequately normalize the distance data. We specified a gaussian distribution and an identity link function for daily range size and distance to bait models and a gamma distribution and a log link function for the distance traveled model. We specified a zero‐inflated beta distribution and a logit link function for the proportion of daily range overlap model, where the beta component modeled non‐zero overlap values and the zero‐inflation component modeled the probability of observing structural zeros. The residuals in the daily range overlap model were overdispersed, so we modeled the dispersion parameter separately from the mean structure by specifying a dispersion formula that included full model structure (Brooks et al. 2017).

We used the *model_parameters* function from the parameters package (v. 0.24.2; Lüdecke et al. 2020) to generate 95% confidence limits for model coefficients. We used the *Anova* function from the car package (v. 3.1‐3; Fox and Weisberg 2019) to perform Type III Wald chi‐square tests, evaluating the significance of each main effect and interaction while controlling for all other terms in the model. We used the *emmeans* function from the emmeans package (v. 1.10.7; Lenth 2025) to calculate estimated marginal means, or least squares means, for pairwise comparisons of mean differences. Estimated marginal means were averaged over the levels of factors that were included in the model but not directly involved in the interpretation (Lenth 2025). For example, when generating pairwise comparisons among bait period levels, results were averaged across levels of sex, treatment, and activity period (Lenth 2025). We used the *cld* function from the multcomp package (v. 1.4‐28; Hothorn et al. 2008) to generate compact letter displays summarizing all pairwise comparisons, which were adjusted for multiple testing using the Sidak‐adjusted pairwise comparisons method. Last, we used the *ggpredict* function from the ggeffects package (v. 2.2.0; Lüdecke 2018) to generate model‐predicted marginal means of space use and movement. These estimates were computed from the fitted models with log or logit links, back‐transformed to the original response scale for interpretation, and accompanied by 95% confidence intervals calculated using the delta method (Lüdecke 2018).

## 3 Results

We analyzed data from 64 GPS collars that met our inclusion criteria, with 41 (20 F, 21 M) and 23 (15 F, 8 M) assigned to the impact and control groups, respectively. Three females (2 impact, one control) were recaptured, resulting in 61 unique individuals (32 F, 29 M). We sampled 18 females and 15 males in 2012, and 17 females and 14 males in 2013. Over the 42‐day study period, the 64 GPS collars collected 256,460 locations with a mean fix success rate of 99.4% ± 0.22 SE. The mean age of deer included in our study was 3.5 years (*n* = 63, SE = 0.2, range = 1.5–6.5).

### 3.1 Range Size

**FIGURE 2:**
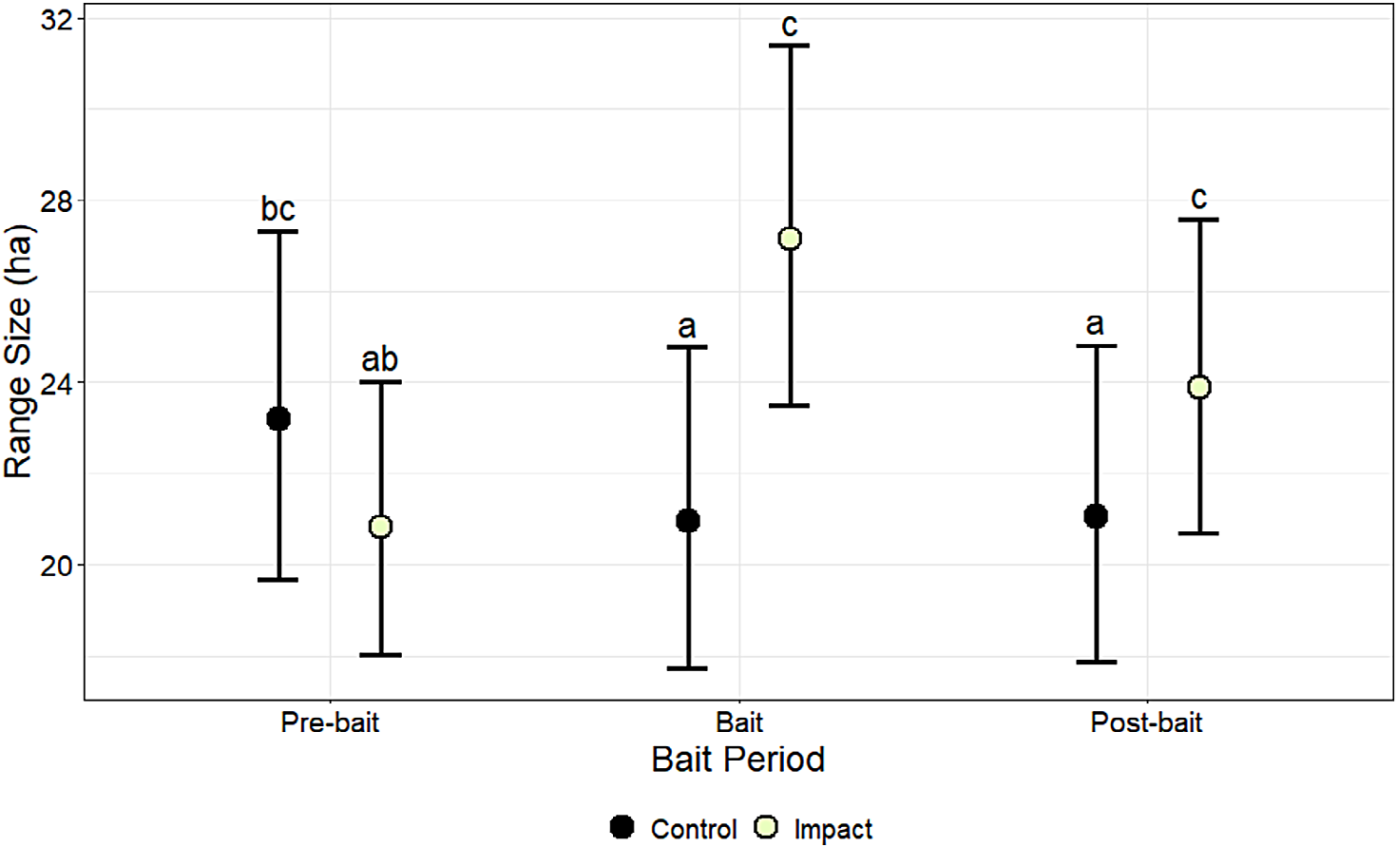
The effects of bait period (pre‐bait, bait, post‐bait) and treatment (control, impact) on model‐predicted estimates of daily range size (ha), during the active period (crepuscular, night), along with associated 95% lower and upper confidence limits, for white‐tailed deer ( *Odocoileus virginianus* ) at Joint Base San Antonio–Camp Bullis, Texas, USA, in 2012–2013. Means with the same letter are not significantly different (*α* > 0.05), based on Sidak‐adjusted pairwise comparisons.

Daily range size (ha/day) during the active period was affected by the interaction between bait period and treatment (*χ* ^2^ = 28.86, df = 2, *p* < 0.001) (Table A1). During the active period, range size was related positively to cover of woody vegetation (*β* = 0.04, *p* = 0.05) (Table A2). During the inactive period, range size was not affected by bait period, sex, treatment, or their interactions (Table A1). During the inactive period, range size was not affected by cover of woody vegetation (*β* = −0.01, *p* = 0.57) (Table A2). Generally, during the active period, daily range size was larger for deer in the impact group, except during the pre‐bait period (Figure 2). When evaluating the interaction between bait period and treatment, during the active period, range size of deer in the impact group increased by 30% (6.3 ha) from the pre‐bait (20.8 ha) to the bait period (27.1 ha) (*p* < 0.001) and by 15% (3.1 ha) from the pre‐bait to the post‐bait period (23.9 ha) (*p* = 0.01), but was not different between the bait and post‐bait periods (*p* > 0.05) (Figure 2; Table A3).

### 3.2 Range Overlap

**FIGURE 3:**
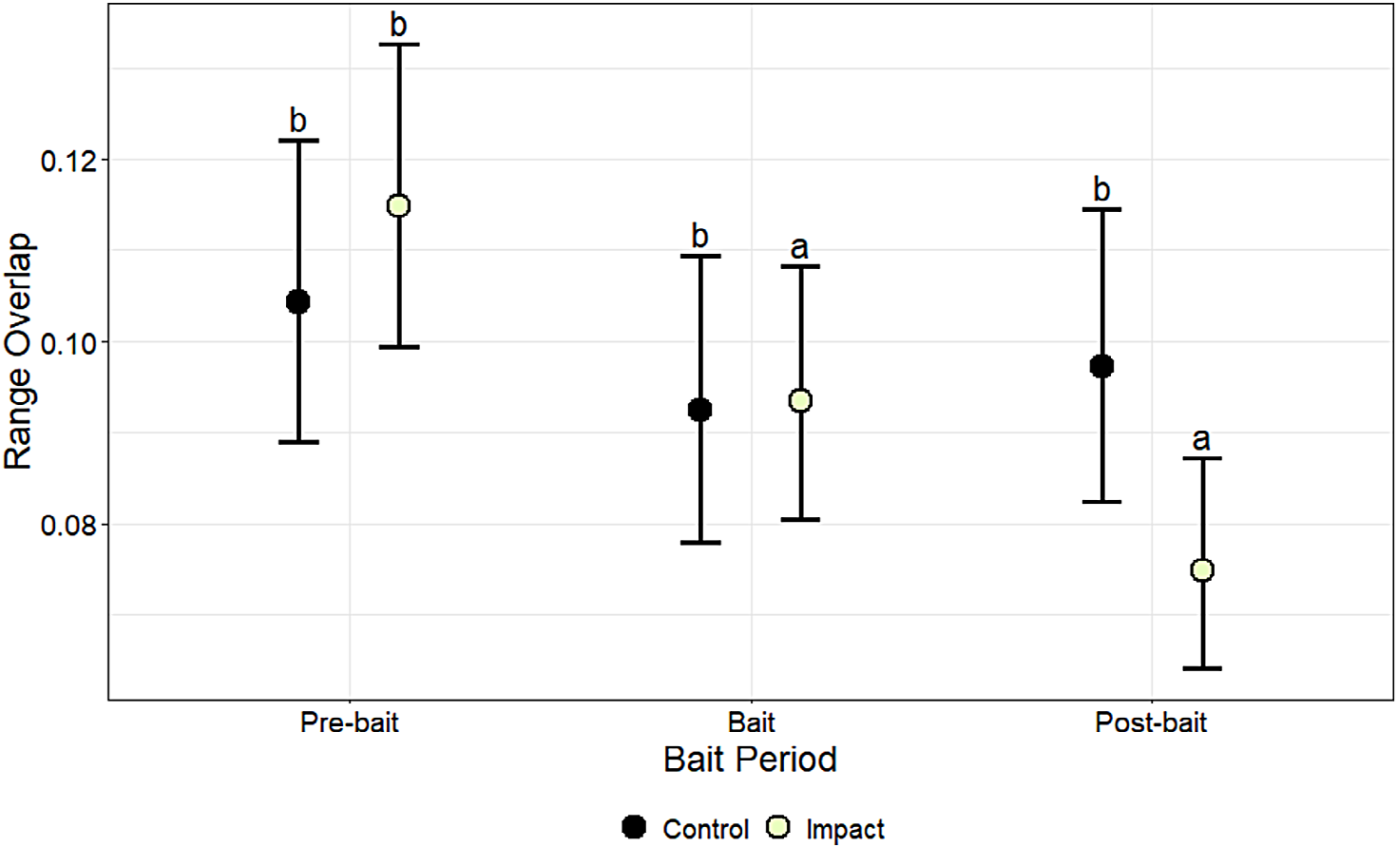
The effects of bait period (pre‐bait, bait, post‐bait) and treatment (control, impact) on model‐predicted estimates of daily range overlap (proportion; 0–1), along with associated 95% lower and upper confidence limits, for white‐tailed deer ( *Odocoileus virginianus* ) at Joint Base San Antonio–Camp Bullis, Texas, USA, in 2012–2013. Means with the same letter are not significantly different (*α* > 0.05), based on Sidak‐adjusted pairwise comparisons.

**FIGURE 4:**
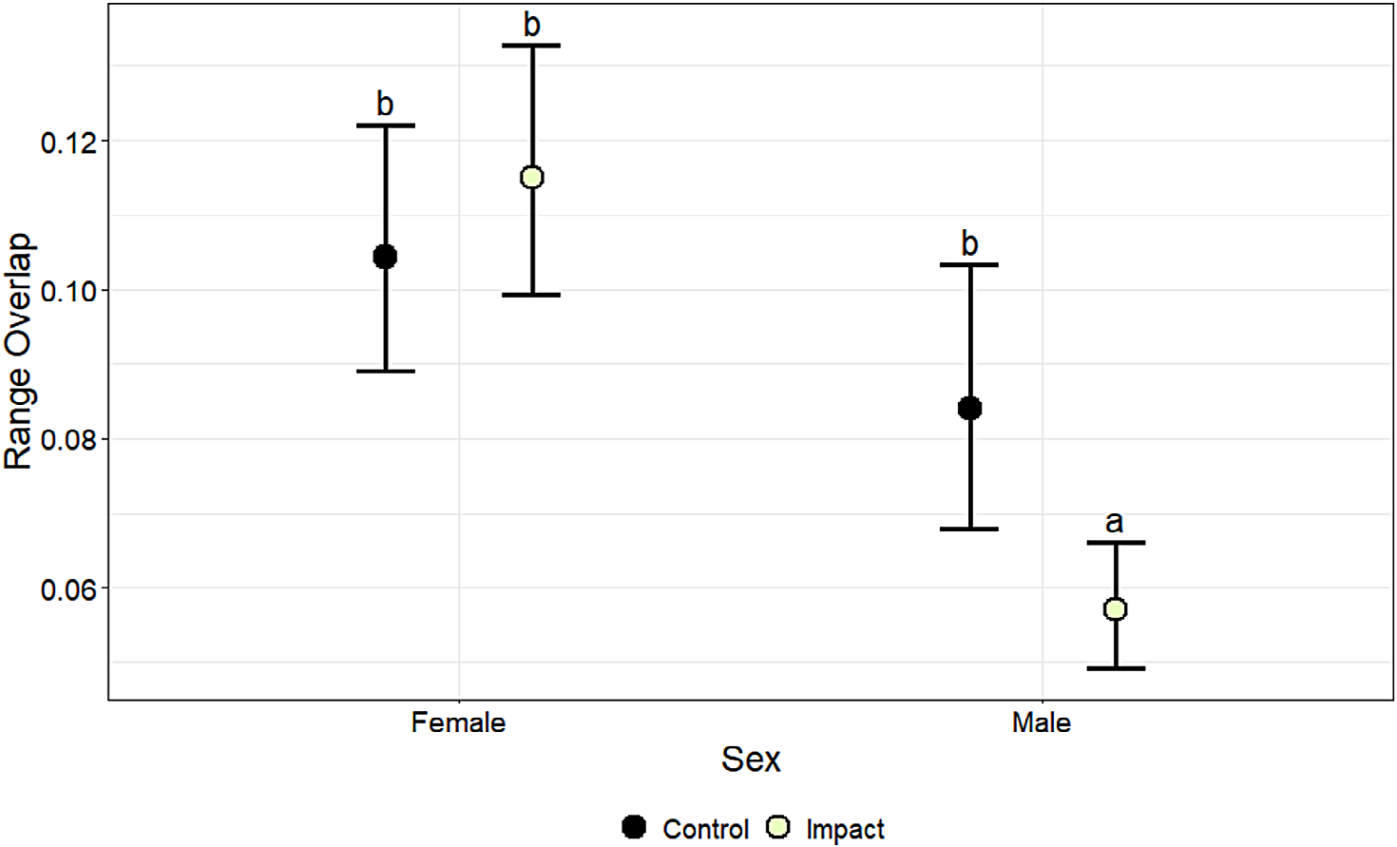
The effects of sex (female, male) and treatment (control, impact) on model‐predicted estimates of daily range overlap (proportion; 0–1), along with associated 95% lower and upper confidence limits, for white‐tailed deer ( *Odocoileus virginianus* ) at Joint Base San Antonio–Camp Bullis, Texas, USA, in 2012–2013. Means with the same letter are not significantly different (*α* > 0.05), based on Sidak‐adjusted pairwise comparisons.

Proportion of range overlap (0–1) was affected by the interaction between bait period and treatment (*χ* ^2^ = 13.04, df = 2, *p* < 0.01) and between sex and treatment (*χ* ^2^ = 8.92, df = 2, *p* < 0.01) (Table A4). Range overlap was not affected by cover of woody vegetation (*β* = 0.00, *p* = 0.91) (Table A5). When evaluating the interaction between bait period and treatment, range overlap of deer in the impact group decreased by 18% (0.02) from the pre‐bait (0.11) to the bait period (0.09) (*p* < 0.001) and by 36% (0.04) from the pre‐bait to the post‐bait period (0.07) (*p* < 0.001), but was not different between the bait and post‐bait periods (*p* > 0.05) (Figure 3; Table A6). When evaluating the interaction between sex and treatment, range overlap of male deer was 25% lower (0.02) in the impact group (0.06) than in the control group (0.08) (*p* < 0.001), whereas range overlap of female deer did not differ between impact and control groups (*p* > 0.05; Figure 4; Table A7).

### 3.3 Distance to Bait

Distance of activity centers to the nearest bait site (m/day) was not affected by bait period, sex, activity period, or their interactions (Table A8). Deer in the impact and control groups maintained mean distances of 386 m (±21 SE) and 2097 m (±280 SE), respectively, between their activity centers and the nearest bait site, consistent with their classification as impact or control groups. The distance of deer activity centers to the nearest bait site was related positively to cover of woody vegetation (*β* = 0.08, *p* < 0.001) (Table A9).

### 3.4 Distance Traveled

**FIGURE 5:**
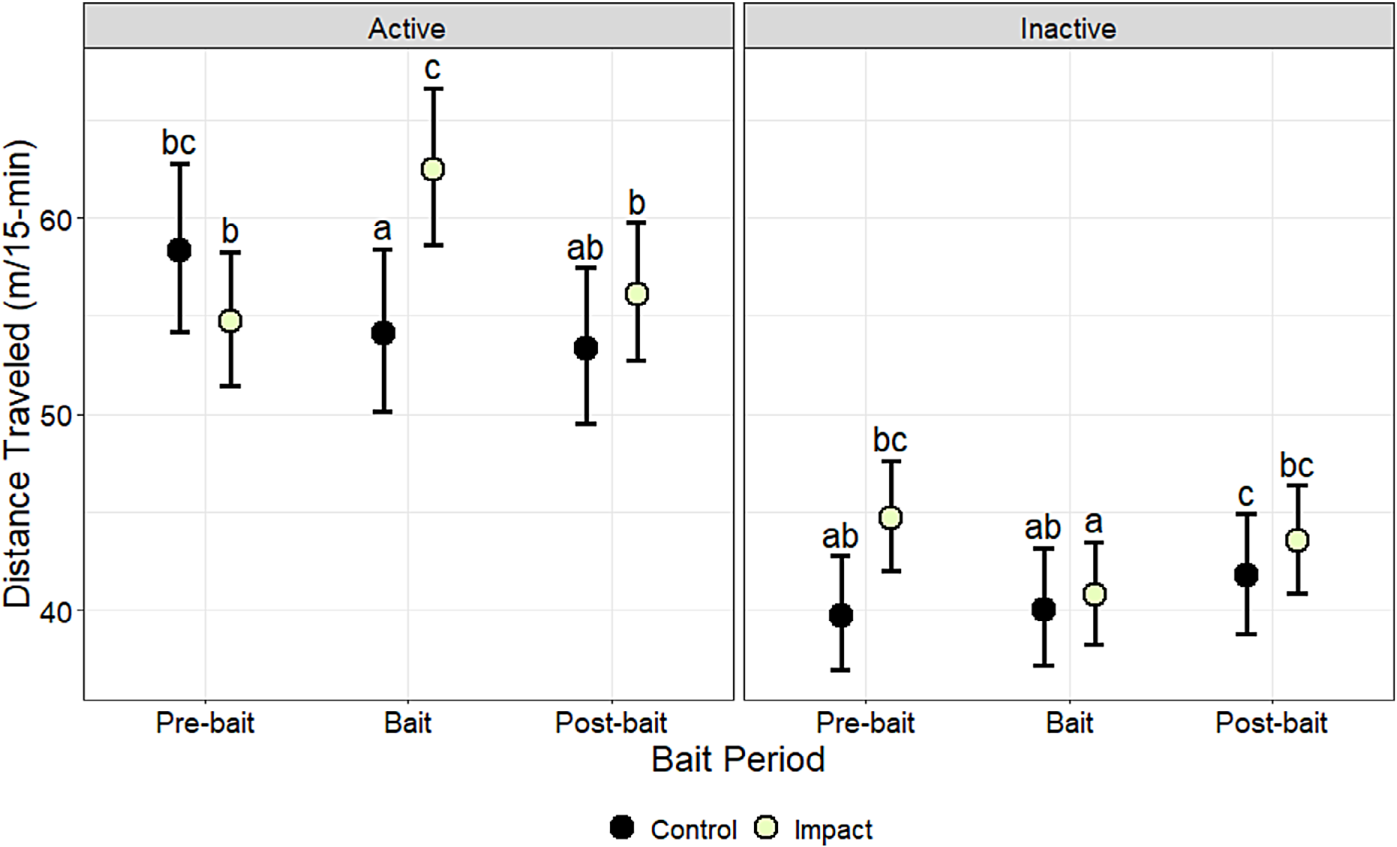
The effects of bait period (pre‐bait, bait, post‐bait), treatment (control, impact), and activity period (active, inactive) on model‐predicted estimates of mean distance traveled (m/15‐min), along with associated 95% lower and upper confidence limits, for white‐tailed deer ( *Odocoileus virginianus* ) at Joint Base San Antonio–Camp Bullis, Texas, USA, in 2012–2013. Sidak‐adjusted pairwise comparisons were conducted within activity periods; means sharing the same letter are not significantly different (*α* > 0.05).

**FIGURE 6:**
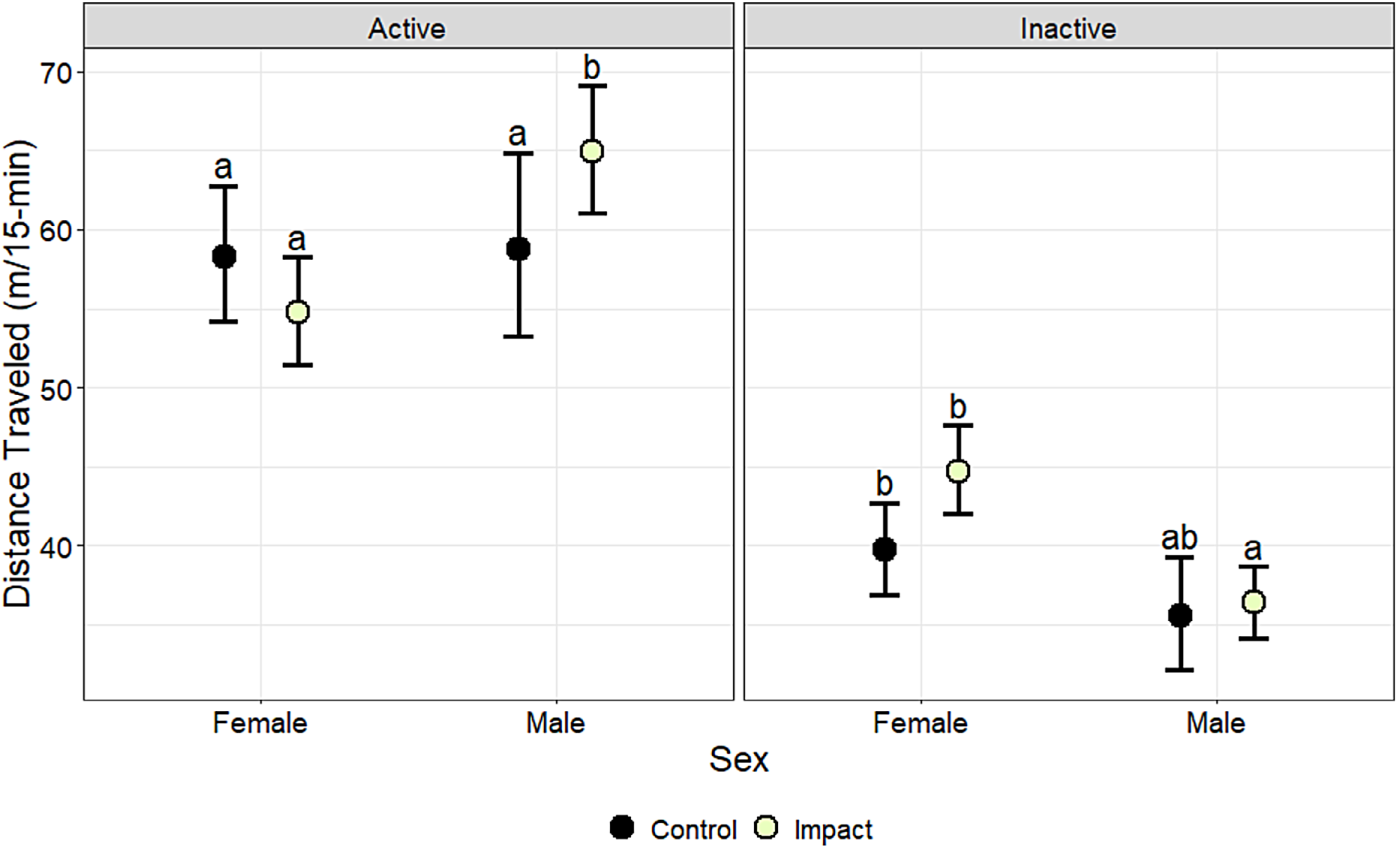
The effects of sex (female, male), treatment (control, impact), and activity period (active, inactive) on model‐predicted estimates of mean distance traveled (m/15‐min), along with associated 95% lower and upper confidence limits, for white‐tailed deer ( *Odocoileus virginianus* ) at Joint Base San Antonio–Camp Bullis, Texas, USA, in 2012–2013. Sidak‐adjusted pairwise comparisons were conducted within activity periods; means sharing the same letter are not significantly different (*α* > 0.05).

Distance traveled (m/15‐min/day) was affected by bait period (*χ* ^2^ = 8.70, df = 2, *p* < 0.01), activity period (*χ* ^2^ = 134.14, df = 1, *p* < 0.001) and the interactions between bait period and treatment (*χ* ^2^ = 22.40, df = 2, *p* < 0.001), sex and treatment (*χ* ^2^ = 4.16, df = 1, *p* = 0.04), bait period and activity period (*χ* ^2^ = 9.35, df = 2, *p* = 0.01), sex and activity period (*χ* ^2^ = 4.76, df = 1, *p* = 0.03), treatment and activity period (*χ* ^2^ = 18.06, df = 1, *p* < 0.001), bait period, treatment, and activity period (*χ* ^2^ = 25.21, df = 2, *p* < 0.001), and sex, treatment, and activity (*χ* ^2^ = 14.76, df = 1, *p* < 0.001) (Table A10). Distance traveled was related positively to cover of woody vegetation (*β* = 0.02, *p* = 0.01) (Table A11). When evaluating the interaction among bait period, treatment, and activity period, during the active period, distance traveled by deer in the impact group increased by 14% (8 m/15 min) from the pre‐bait (55 m/15 min) to the bait period (62 m/15 min) (*p* < 0.01) and decreased by 10% (6 m/15 min) from the bait to the post‐bait period (56 m/15 min) (*p* = 0.03) but was not statistically different between the pre‐bait and post‐bait (*p* > 0.05) (Figure 5; Table A12). Conversely, when evaluating the same interaction, during the inactive period, distance traveled by deer in the impact group decreased by 9% (4 m/15 min) from the pre‐bait (45 m/15 min) to the bait period (41 m/15 min) (*p* < 0.001) and increased by 7% (3 m/15 min) from the bait to the post‐bait period (44 m/15 min) (*p* < 0.001) but was not statistically different between the pre‐bait and post‐bait (*p* > 0.05) (Figure 5; Table A12). When evaluating the interaction among sex, treatment, and activity period, distance traveled by male deer during the active period was 10% (6 m/15 min) greater in the impact group (65 m/15 min) compared to the control group (59 m/15 min) (*p* < 0.01) (Figure 6; Table A13).

## 4 Discussion

We observed spatial responses of deer to the presence of temporary bait, especially when compared to a control group of deer without bait sites within their home ranges (i.e., through the use of a BACI design). When bait was present, deer exposed to temporary bait (impact group) exhibited larger range sizes and greater movement during active periods but showed reduced movement during inactive periods relative to the pre‐bait period, highlighting time‐dependent behavioral responses to baiting. Contrary to previous studies, we did not detect a spatial shift of deer activity centers toward bait sites. Support for our prediction that deer would exhibit search behavior (i.e., increased movement, larger ranges, and reduced range overlap) following bait removal was limited, and the effects detected were relatively small. Although several predictors were statistically significant, variation in effect magnitudes complicated interpretation of their biological and management relevance.

Temporary bait is often used to attract deer to targeted sites for camera surveys and harvest management. Our results show that, during the active period, deer with access to baited sites within their home ranges exhibited greater movement and larger ranges compared to deer in the control group. However, deer did not shift their activity centers closer to bait sites once bait was available, which contrasts with previous studies reporting that deer may adjust their centers of activity in response to the presence or removal of bait (Williams and DeNicola 2000; Kilpatrick and Stober 2002; Campbell et al. 2006; Rustand 2010). For example, Campbell et al. (2006) and Williams and DeNicola (2000) observed shifts of 83 m (15%) and 115 m, respectively, toward baited locations, while Vercauteren and Hygnstrom (1998) reported deer moving 174 m closer to cornfields during crop maturation. This discrepancy may be partly explained by forage availability; deer are likely to reduce use of supplemental feed during periods of green‐up or mast production (Roden‐Reynolds et al. 2022), decreasing visitation to bait and feeder sites. Notably, our study coincided with record‐high precipitation in May 2012 and 2013 (NOAA 2023), resulting in abundant forage, which may have diminished the effect of bait presence and removal on space use and movements. Another consideration is bait or crop exposure duration: previous studies involved 21–99 days of exposure (Campbell et al. 2006, 2012; Williams and DeNicola 2000; Vercauteren and Hygnstrom 1998; Kilpatrick and Stober 2002), far exceeding the 11‐ and 14‐day baiting periods used in our study, which may have reduced the likelihood of resource dependency. Collectively, these findings suggest that temporary bait is unlikely to attract deer outside established ranges when forage production is high and baiting is brief. Future research should examine the effects of temporary bait under conditions of limited or declining forage availability and across varying durations of exposure, from short‐term (days to weeks) to long‐term (months).

Another concern with using bait is that its removal may trigger search behavior in deer, manifested as increased movement distances, larger range sizes, and reduced range overlap (site fidelity), potentially driving them across property boundaries and increasing their risk, particularly when bait is present during hunting season. We found little evidence supporting our prediction that deer would exhibit search behavior following bait removal. The limited support was characterized by small effect sizes that are unlikely to be biologically meaningful or relevant for management. For example, among deer in the impact group, range size during the active period was 15% greater and range overlap 36% lower in the post‐bait period compared to the pre‐bait period. However, no changes in these metrics were observed from the bait to the post‐bait period when search behavior would be expected. Our findings on space use contrast those of Campbell et al. (2012), who reported that wild pig ( *Sus scrofa* ) home ranges expanded by 103% following the removal of whole kernel corn, and with Vercauteren and Hygnstrom (1998), who found that female white‐tailed deer home ranges increased by 32% after corn harvest as deer sought alternative food and cover resources. In our study, deer exposed to temporary bait did not appear to abandon the area in search of new resources; instead, deer continued to use areas within previously established ranges. Again, we suggest that the discrepancy between our findings and previous studies is, at least partly, due to high forage availability across the landscape and the brief duration of bait exposure, which likely mitigated the effects of bait presence and removal on deer space use and movements. Therefore, conducting baited camera surveys during periods of high food abundance may reduce detection rates, but as a tradeoff, deer may be less likely to leave the property in search of food. Likewise, short‐duration camera surveys (≤ 14 days) may help mitigate resource dependency and its effects on space use and movement. Wildlife managers should carefully consider these tradeoffs when designing baited camera surveys.

Activity patterns strongly influence how and when wildlife interact with their environment. In our study, deer responses to the presence and removal of bait varied by time of day, often exhibiting inverse patterns between activity periods or effects that were restricted to a single period. Specifically, during the active period, deer in the impact group exhibited increased movements and larger range sizes from the pre‐bait to the bait period. In contrast, an inverse pattern in movements was observed during the inactive period, when deer typically rest for extended durations, and no effect was detected in range size, suggesting that deer exploited these resources when human activity was minimal and perceived risk was likely lower. Had we not accounted for activity patterns, the opposing trends between active and inactive periods would likely have obscured the effect of bait presence on range size and movements. These results underscore the importance of incorporating activity patterns into analyses of animal space use and behavior.

Our BACI study design, featuring a robust sample size, balanced sex ratio, fine‐scale location data collected at 15‐min intervals (closely matching the time deer spend at feeders), and inclusion of both activity periods, enhanced our ability to evaluate the effects of temporary bait presence and removal. The ability to detect the effects of bait or food sources on deer space use and movement may depend on the frequency of location data collection. Camera surveys at bait sites indicate that the average deer visit lasts < 15 min (Ozoga and Verme 1982; Kozicky 1997; Newbolt et al. 2017). Consequently, infrequent sampling might fail to capture bait‐use events in the location data. Previous studies used VHF telemetry or GPS collected location data at intervals ranging from 30 min (Garver 2011) to 2 h (Rustand 2010), 4 h (Kilpatrick and Stober 2002), or even coarser (Williams and DeNicola 2000; Campbell et al. 2006; Cooper et al. 2006; Webb, Hewitt, et al. 2008). In contrast, we collected deer locations every 15 min, matching typical visit durations at bait sites, to minimize the risk of over‐ or underestimating bait effects on deer spatial distribution and movements. We recommend tailoring GPS‐collar fix intervals to maximize detection of rare events, such as fence crossings, animal contacts, or bait use.

Our findings provide insight into the effects of the presence and removal of temporary bait on deer space use and movements; however, we caution that our findings are likely context‐specific and may be more local in scope. Deer space use and movement patterns often change during hunting (Little et al. 2016) and breeding seasons (Beier and McCullough 1990; Webb et al. 2009) and are influenced by nutritional demands. Our survey, conducted between the fawning and mating seasons (TPWD, n.d.), may not reflect baiting practices in other regions. Seasonal nutritional needs, such as recovery from nursing fawns or antler growth, as well as forage availability and quality, may have shaped how deer responded to temporary bait. Outcomes may also differ when supplemental feed is available for longer durations or at different times of year. Given the brief period that bait was available in our study, deer did not substantially alter their spatial distribution to incorporate this temporary resource within their range if it was not already present. Future research should examine the effects of long‐term baiting or supplemental feeding on deer space use and movement (Johnson et al. 2021), particularly in regions where year‐round feeding is permitted or protein supplementation is common (e.g., in Texas).

## Author Contributions

**Dylan G. Stewart:** conceptualization (equal), data curation (equal), formal analysis (lead), investigation (equal), methodology (equal), software (lead), validation (lead), visualization (lead), writing – original draft (lead), writing – review and editing (equal). **Jared T. Beaver:** conceptualization (equal), data curation (equal), investigation (equal), methodology (equal), project administration (equal), supervision (equal), writing – review and editing (supporting). **M. Lucas Cooksey:** conceptualization (equal), funding acquisition (equal), methodology (equal), project administration (equal), resources (equal), supervision (equal), writing – review and editing (supporting). **Chad Grantham:** conceptualization (equal), funding acquisition (equal), methodology (equal), project administration (equal), resources (equal), supervision (equal), writing – review and editing (supporting). **Brian L. Pierce:** conceptualization (equal), data curation (equal), funding acquisition (equal), methodology (equal), project administration (equal), resources (equal), supervision (equal), writing – review and editing (supporting). **Roel R. Lopez:** conceptualization (equal), funding acquisition (equal), methodology (equal), project administration (equal), resources (equal), supervision (equal), writing – review and editing (supporting). **Stephen L. Webb:** conceptualization (equal), data curation (equal), investigation (equal), methodology (equal), supervision (equal), writing – original draft (supporting), writing – review and editing (equal).

## Funding

This work was supported by the United States Army Corps of Engineers.

## Conflicts of Interest

The authors declare no conflicts of interest.

## Data Availability

The data supporting our findings are publicly available in the Dryad Digital Repository at https://doi.org/10.5061/dryad.wm37pvn2f.

## Appendix A

**TABLE A1:** Wald Chi‐squared statistics (*χ* ^2^), degrees of freedom (df), and probability value (*p*) evaluating the significance of fixed effects (bait period [pre‐bait, bait, post‐bait], sex [female, male], treatment [impact, control], percent woody cover) and their interactions (bait period × sex × treatment) on daily range size (ha) of white‐tailed deer ( *Odocoileus virginianus* ), during the active (crepuscular, night) and inactive (day) periods, at Joint Base San Antonio–Camp Bullis, Texas, USA, during 2012–2013.

Variables	*χ* ^2^	df	*p*
Active period			
Intercept	1379.32	1	< 0.001
Bait period	4.87	2	0.09
Sex	1.38	1	0.24
Treatment	1.11	1	0.29
Woody cover	3.79	1	0.05
Bait period × Sex	1.52	2	0.47
Bait period × Treatment	28.86	2	< 0.001
Sex × Treatment	2.63	1	0.10
Bait period × Sex × Treatment	0.66	2	0.72
Inactive period			
Intercept	650.36	1	< 0.001
Bait period	0.40	2	0.82
Sex	2.05	1	0.15
Treatment	2.12	1	0.14
Woody cover	0.33	1	0.57
Bait period × Sex	3.89	2	0.14
Bait period × Treatment	3.07	2	0.22
Sex × Treatment	0.60	1	0.44
Bait period × Sex × Treatment	0.45	2	0.80

**TABLE A2:** Mean of the log estimates (*β*), standard errors (SE), 95% lower (LCL) and upper (UCL) confidence limits, z score (*z*), and probability value (*p*) predicting the effects of bait period (BP; pre‐bait [PreB], bait [B], post‐bait [PtB]), sex (female [F], male [M]), treatment (TRT; control [C], impact [I]), and percent woody cover, the interaction between bait period, sex, and treatment, and random effects for unique collar event and year on daily range size (ha) of white‐tailed deer ( *Odocoileus virginianus* ), during the active (crepuscular, night) and inactive (day) periods, on Joint Base San Antonio‐Camp Bullis, Texas, USA, in 2012–2013.

Variables	*β*	SE	LCL	UCL	*z*	*p*	Var/SD
Active period							
Intercept	3.16	0.09	3.00	3.33	37.14	< 0.001	
BP (B)	−0.10	0.05	−0.21	0.00	−1.90	0.06	
BP (PtB)	−0.10	0.05	−0.20	0.00	−1.90	0.06	
Sex (M)	0.16	0.13	−0.11	0.42	1.17	0.24	
TRT (I)	−0.11	0.10	−0.31	0.09	−1.05	0.29	
Percent woody	0.04	0.02	0.00	0.08	1.95	0.05	
BP (B) × Sex (M)	−0.10	0.09	−0.27	0.08	−1.10	0.27	
BP (PtB) × Sex (M)	−0.09	0.09	−0.26	0.08	−1.02	0.31	
BP (B) × TRT (I)	0.37	0.07	0.23	0.50	5.26	< 0.001	
BP (PtB) × TRT (I)	0.23	0.07	0.10	0.37	3.49	< 0.001	
Sex (M) × TRT (I)	0.27	0.17	−0.06	0.60	1.62	0.11	
BP (B) × Sex (M) × TRT (I)	−0.06	0.11	−0.28	0.15	−0.59	0.55	
BP (PtB) × Sex (M) × TRT (I)	0.02	0.11	−0.19	0.23	0.20	0.84	
Collar identifier							0.07/0.26
Year							0.00/0.05
Inactive period							
Intercept	2.03	0.08	1.87	2.19	25.50	< 0.001	
BP (B)	−0.04	0.08	−0.20	0.11	−0.57	0.57	
BP (PtB)	0.00	0.07	−0.15	0.14	−0.04	0.97	
Sex (M)	−0.19	0.14	−0.46	0.07	−1.43	0.15	
TRT (I)	0.15	0.11	−0.05	0.36	1.46	0.15	
Percent woody	−0.01	0.02	−0.05	0.03	−0.57	0.57	
BP (B) × Sex (M)	−0.03	0.13	−0.28	0.22	−0.23	0.81	
BP (PtB) × Sex (M)	0.20	0.13	−0.05	0.44	1.60	0.11	
BP (B) × TRT (I)	−0.18	0.10	−0.37	0.02	−1.75	0.08	
BP (PtB) × TRT (I)	−0.07	0.10	−0.26	0.12	−0.75	0.45	
Sex (M) × TRT (I)	−0.13	0.17	−0.45	0.20	−0.77	0.44	
BP (B) × Sex (M) × TRT (I)	−0.08	0.16	−0.39	0.23	−0.49	0.63	
BP (PtB) × Sex (M) × TRT (I)	−0.10	0.15	−0.40	0.20	−0.64	0.52	
Collar identifier							0.05/0.23
Year							0.00/0.00

**TABLE A3:** Model‐predicted marginal mean estimates ($\hat{y}$) of daily range size (ha) of white‐tailed deer ( *Odocoileus virginianus* ), with corresponding 95% lower (LCL) and upper (UCL) confidence limits, by bait period (pre‐bait, bait, post‐bait) and treatment (control, impact), during the active (crepuscular, night) period, at Joint Base San Antonio–Camp Bullis, Texas, USA, 2012–2013.

Activity period	Bait period	Treatment					
		Control			Impact		
		$\hat{y}$	LCL	UCL	$\hat{y}$	LCL	UCL
Active	Pre‐bait	23.18	19.68	27.31	20.81	18.02	24.03
	Bait	20.96	17.74	24.77	27.14	23.47	31.38
	Post‐bait	21.05	17.86	24.82	23.89	20.69	27.58

**TABLE A4:** Wald Chi‐squared statistics (*χ* ^2^), degrees of freedom (df), and probability value (*p*) evaluating the significance of fixed effects (bait period [pre‐bait, bait, post‐bait], sex [female, male], treatment [impact, control], activity period [active, inactive], percent woody cover) and their interactions (bait period × sex × treatment × activity period) on daily range overlap (proportion; 0–1) of white‐tailed deer ( *Odocoileus virginianus* ) at Joint Base San Antonio–Camp Bullis, Texas, USA, during 2012–2013.

Variables	*χ* ^2^	df	*p*
Intercept	635.93	1	< 0.001
Bait period	2.69	2	0.26
Sex	3.00	1	0.08
Treatment	1.00	1	0.32
Activity period	1.15	1	0.28
Woody cover	0.01	1	0.91
Bait period × Sex	2.72	2	0.26
Bait period × Treatment	13.04	2	< 0.01
Sex × Treatment	8.92	1	< 0.01
Bait period × Activity period	1.26	2	0.53
Sex × Activity period	3.51	1	0.06
Treatment × Activity period	0.47	1	0.49
Bait period × Sex × Treatment	2.38	2	0.30
Bait period × Sex × Activity period	2.01	2	0.37
Bait period × Treatment × Activity period	5.57	2	0.06
Sex × Treatment × Activity	3.26	1	0.07
Bait period × Sex × Treatment × Activity period	5.14	2	0.08

**TABLE A5:** Mean of the logit estimates (*β*), standard errors (SE), 95% lower (LCL) and upper (UCL) confidence limits, z score (*z*), and probability value (*p*) predicting the effects of bait period (BP; pre‐bait [PreB], bait [B], post‐bait [PtB]), sex (female [F], male [M]), treatment (TRT; control [C], impact [I]), activity period (AP; active [Act], inactive [InAc]), and percent woody cover, the interaction between bait period, sex, treatment, and activity period, and random effects for unique collar event and year on daily range overlap (proportion; 0–1) of white‐tailed deer ( *Odocoileus virginianus* ) on Joint Base San Antonio‐Camp Bullis, Texas, USA, in 2012–2013.

Variables	*β*	SE	LCL	UCL	*z*	*p*	Var/SD
Intercept	−2.15	0.09	−2.31	−1.98	−25.22	< 0.001	
BP (B)	−0.13	0.08	−0.30	0.03	−1.62	0.11	
BP (PB)	−0.08	0.08	−0.23	0.08	−0.98	0.33	
Sex (M)	−0.24	0.14	−0.51	0.03	−1.73	0.08	
Treatment (I)	0.11	0.11	−0.10	0.32	1.00	0.32	
AP (InAc)	0.08	0.08	−0.07	0.24	1.07	0.28	
Woody cover	0.00	0.02	−0.04	0.03	−0.11	0.91	
BP (B) × Sex (M)	0.20	0.14	−0.09	0.48	1.35	0.18	
BP (PB) × Sex (M)	0.22	0.15	−0.08	0.52	1.45	0.15	
BP (B) × TRT (I)	−0.10	0.12	−0.32	0.13	−0.85	0.40	
BP (PB) × TRT (I)	−0.39	0.11	−0.62	−0.17	−3.49	< 0.001	
Sex (M) × TRT (I)	−0.52	0.18	−0.87	−0.18	−2.99	< 0.01	
BP (B) × AP (InAc)	0.05	0.12	−0.18	0.27	0.39	0.70	
BP (PB) × AP (InAc)	−0.08	0.11	−0.30	0.13	−0.76	0.45	
Sex (M) × AP (InAc)	−0.26	0.14	−0.52	0.01	−1.87	0.06	
Treatment (I) × AP (InAc)	0.07	0.11	−0.14	0.28	0.69	0.49	
BP (B) × Sex (M) × TRT (I)	−0.05	0.18	−0.41	0.30	−0.29	0.77	
BP (PB) × Sex (M) × TRT (I)	0.23	0.19	−0.14	0.60	1.22	0.22	
BP (B) × Sex (M) × AP (InAc)	−0.01	0.21	−0.42	0.39	−0.07	0.95	
BP (PB) × Sex (M) × AP (InAc)	0.26	0.21	−0.15	0.67	1.24	0.21	
BP (B) × TRT (I) × AP (InAc)	−0.12	0.16	−0.43	0.19	−0.73	0.46	
BP (PB) × TRT (I) × AP (InAc)	0.25	0.15	−0.05	0.55	1.63	0.10	
Sex (M) × TRT (I) × AP (InAc)	0.31	0.17	−0.03	0.64	1.81	0.07	
BP (B) × Sex (M) × TRT (I) × AP (InAc)	−0.04	0.25	−0.54	0.45	−0.17	0.87	
BP (PB) × Sex (M) × TRT (I) × AP (InAc)	−0.53	0.25	−1.03	−0.03	−2.10	0.04	
Collar identifier							0.04/0.21
Year							0.00/0.05

**TABLE A6:** Model‐predicted marginal mean estimates ($\hat{y}$) of daily range overlap (0–1) of white‐tailed deer ( *Odocoileus virginianus* ), with corresponding 95% lower (LCL) and upper (UCL) confidence limits, by bait period (pre‐bait, bait, post‐bait) and treatment (control, impact) at Joint Base San Antonio–Camp Bullis, Texas, USA, 2012–2013.

Bait period	Treatment					
	Control			Impact		
	$\hat{y}$	LCL	UCL	$\hat{y}$	LCL	UCL
Pre‐bait	0.10	0.09	0.12	0.11	0.10	0.13
Bait	0.09	0.08	0.11	0.09	0.08	0.11
Post‐bait	0.10	0.08	0.11	0.07	0.06	0.09

**TABLE A7:** Model‐predicted marginal mean estimates ($\hat{y}$) of daily range overlap (0–1) of white‐tailed deer ( *Odocoileus virginianus* ), with corresponding 95% lower (LCL) and upper (UCL) confidence limits, by sex (female, male) and treatment (control, impact) at Joint Base San Antonio–Camp Bullis, Texas, USA, 2012–2013.

Sex	Treatment					
	Control			Impact		
	$\hat{y}$	LCL	UCL	$\hat{y}$	LCL	UCL
Female	0.10	0.09	0.12	0.11	0.10	0.13
Male	0.08	0.07	0.10	0.06	0.05	0.07

**TABLE A8:** Wald Chi‐squared statistics (*χ* ^2^), degrees of freedom (df), and probability value (*p*) evaluating the significance of fixed effects (bait period [pre‐bait, bait, post‐bait], sex [female, male], activity period [active, inactive], percent woody cover) and their interactions (bait period × sex × activity period) on distance (m) of activity centers to the nearest bait site of white‐tailed deer ( *Odocoileus virginianus* ) at Joint Base San Antonio–Camp Bullis, Texas, USA, during 2012–2013.

Variables	*χ* ^2^	df	*p*
Intercept	1.20	1	0.27
Bait period	3.60	2	0.17
Sex	1.66	1	0.20
Activity period	1.36	1	0.24
Woody cover	59.41	1	< 0.001
Bait period × Sex	2.17	2	0.34
Bait period × Activity period	1.75	2	0.42
Sex × Activity period	2.60	1	0.11
Bait period × Sex × Activity period	0.38	2	0.83

**TABLE A9:** Normalized mean estimates (*β*), standard errors (SE), 95% lower (LCL) and upper (UCL) confidence limits, z score (*z*), and probability value (*p*) predicting the effects of bait period (BP; pre‐bait [PreB], bait [B], post‐bait [PtB]), sex (female [F], male [M]), activity period (AP; active [Act], inactive [InAc]), and percent woody cover, the interaction between bait period, sex, and activity period, and random effects for unique collar event and year on distance (m) of activity centers to the nearest bait site of white‐tailed deer ( *Odocoileus virginianus* ) on Joint Base San Antonio‐Camp Bullis, Texas, USA, in 2012–2013.

Variables	*β*	SE	LCL	UCL	*z*	*p*	Var/SD
Intercept	0.16	0.15	−0.13	0.45	1.09	0.27	
BP (B)	−0.02	0.03	−0.09	0.04	−0.71	0.48	
BP (PtB)	0.04	0.03	−0.02	0.10	1.20	0.23	
Sex (M)	−0.28	0.22	−0.71	0.15	−1.29	0.20	
AP (Act)	−0.04	0.03	−0.10	0.03	−1.17	0.24	
Percent woody	0.08	0.01	0.06	0.10	7.71	< 0.001	
BP (B) × Sex (M)	0.06	0.05	−0.03	0.15	1.27	0.20	
BP (PtB) × Sex (M)	0.00	0.05	−0.09	0.09	−0.04	0.97	
BP (B) × AP (Act)	−0.06	0.05	−0.15	0.03	−1.29	0.20	
BP (PtB) × AP (Act)	−0.04	0.04	−0.13	0.05	−0.89	0.37	
Sex (M) × AP (Act)	−0.07	0.05	−0.17	0.02	−1.61	0.11	
BP (B) × Sex (M) × AP (Act)	0.02	0.07	−0.11	0.15	0.34	0.74	
BP (PtB) × Sex (M) × AP (Act)	0.04	0.07	−0.09	0.17	0.62	0.54	
Collar identifier							0.74/0.86
Year							0.00/0.00

**TABLE A10:** Wald Chi‐squared statistics (*χ* ^2^), degrees of freedom (df), and probability value (*p*) evaluating the significance of fixed effects (bait period [pre‐bait, bait, post‐bait], sex [female, male], treatment [impact, control], activity period [active, inactive], percent woody cover) and their interactions (bait period × sex × treatment × activity period) on mean distance traveled (m/15‐min) of white‐tailed deer ( *Odocoileus virginianus* ) at Joint Base San Antonio–Camp Bullis, Texas, USA, during 2012–2013.

Variables	*χ* ^2^	df	*p*
Intercept	11,591.75	1	< 0.001
Bait period	8.70	2	0.01
Sex	0.01	1	0.91
Treatment	1.68	1	0.19
Activity period	134.14	1	< 0.001
Woody cover	6.88	1	0.01
Bait period × Sex	2.47	2	0.29
Bait period × Treatment	22.40	2	< 0.001
Sex × Treatment	4.16	1	0.04
Bait period × Activity period	9.35	2	0.01
Sex × Activity period	4.76	1	0.03
Treatment × Activity period	18.06	1	< 0.001
Bait period × Sex × Treatment	0.80	2	0.67
Bait period × Sex × Activity period	2.13	2	0.34
Bait period × Treatment × Activity period	25.21	2	< 0.001
Sex × Treatment × Activity	14.76	1	< 0.001
Bait period × Sex × Treatment × Activity period	0.00	2	1.00

**TABLE A11:** Mean of the log estimates (*β*), standard errors (SE), 95% lower (LCL) and upper (UCL) confidence limits, z score (*z*), and probability value (*p*) predicting the effects of bait period (BP; pre‐bait [PreB], bait [B], post‐bait [PtB]), sex (female [F], male [M]), treatment (TRT; control [C], impact [I]), activity period (AP; active [Act], inactive [InAc]), and percent woody cover, the interaction between bait period, sex, treatment, and activity period, and random effects for unique collar event and year on mean distance traveled (m/15‐min) of white‐tailed deer ( *Odocoileus virginianus* ) on Joint Base San Antonio‐Camp Bullis, Texas, USA, in 2012–2013.

Variables	*β*	SE	LCL	UCL	*z*	*p*	Var/SD
Intercept	4.07	0.04	3.99	4.14	107.67	< 0.001	
BP (B)	−0.07	0.03	−0.14	−0.01	−2.23	0.03	
BP (PB)	−0.09	0.03	−0.15	−0.03	−2.76	0.01	
Sex (M)	0.01	0.06	−0.12	0.13	0.12	0.91	
Treatment (I)	−0.06	0.05	−0.16	0.03	−1.30	0.20	
AP (InAc)	−0.38	0.03	−0.45	−0.32	−11.58	< 0.001	
Woody cover	0.02	0.01	0.00	0.03	2.62	0.01	
BP (B) × Sex (M)	−0.05	0.06	−0.16	0.06	−0.96	0.34	
BP (PB) × Sex (M)	0.03	0.05	−0.07	0.14	0.62	0.54	
BP (B) × TRT (I)	0.21	0.04	0.12	0.29	4.71	< 0.001	
BP (PB) × TRT (I)	0.11	0.04	0.03	0.20	2.69	0.01	
Sex (M) × TRT (I)	0.16	0.08	0.01	0.32	2.04	0.04	
BP (B) × AP (InAc)	0.08	0.05	−0.01	0.18	1.74	0.08	
BP (PB) × AP (InAc)	0.14	0.05	0.05	0.23	3.04	< 0.01	
Sex (M) × AP (InAc)	−0.12	0.05	−0.23	−0.01	−2.18	0.03	
Treatment (I) × AP (InAc)	0.18	0.04	0.10	0.27	4.25	< 0.001	
BP (B) × Sex (M) × TRT (I)	−0.05	0.07	−0.19	0.08	−0.79	0.43	
BP (PB) × Sex (M) × TRT (I)	−0.05	0.07	−0.18	0.08	−0.74	0.46	
BP (B) × Sex (M) × AP (InAc)	0.07	0.08	−0.08	0.23	0.89	0.37	
BP (PB) × Sex (M) × AP (InAc)	0.11	0.08	−0.04	0.26	1.44	0.15	
BP (B) × TRT (I) × AP (InAc)	−0.31	0.06	−0.43	−0.19	−4.94	< 0.001	
BP (PB) × TRT (I) × AP (InAc)	−0.19	0.06	−0.31	−0.07	−3.16	< 0.01	
Sex (M) × TRT (I) × AP (InAc)	−0.26	0.07	−0.39	−0.13	−3.84	< 0.001	
BP (B) × Sex (M) × TRT (I) × AP (InAc)	0.00	0.10	−0.19	0.19	0.02	0.98	
BP (PB) × Sex (M) × TRT (I) × AP (InAc)	0.00	0.09	−0.18	0.19	0.01	0.99	
Collar identifier							0.01/0.11
Year							0.00/0.01

**TABLE A12:** Model‐predicted marginal mean estimates ($\hat{y}$) of mean distance traveled (m/15‐m) of white‐tailed deer ( *Odocoileus virginianus* ), with corresponding 95% lower (LCL) and upper (UCL) confidence limits, by bait period (pre‐bait, bait, post‐bait), treatment (control, impact), and activity period (active, inactive) at Joint Base San Antonio–Camp Bullis, Texas, USA, 2012–2013.

Activity period	Bait period	Treatment					
		Control			Impact		
		$\hat{y}$	LCL	UCL	$\hat{y}$	LCL	UCL
Active	Pre‐bait	58.31	54.15	62.79	54.73	51.40	58.28
	Bait	54.10	50.11	58.41	62.49	58.62	66.62
	Post‐bait	53.35	49.53	57.46	56.13	52.71	59.78
Inactive	Pre‐bait	39.73	36.94	42.73	44.70	41.98	47.60
	Bait	40.04	37.14	43.16	40.76	38.22	43.47
	Post‐bait	41.74	38.81	44.90	43.52	40.87	46.36

**TABLE A13:** Model‐predicted marginal mean estimates ($\hat{y}$) of mean distance traveled (m/15‐m) of white‐tailed deer ( *Odocoileus virginianus* ), with corresponding 95% lower (LCL) and upper (UCL) confidence limits, by sex (female, male), treatment (control, impact), and activity period (active, inactive) at Joint Base San Antonio–Camp Bullis, Texas, USA, 2012–2013.

Activity period	Sex	Treatment					
		Control			Impact		
		$\hat{y}$	LCL	UCL	$\hat{y}$	LCL	UCL
Active	Female	58.31	54.15	62.79	54.73	51.40	58.28
	Male	58.76	53.21	64.88	64.91	61.00	69.08
Inactive	Female	39.73	36.94	42.73	44.70	41.98	47.60
	Male	35.54	32.14	39.30	36.37	34.17	38.71
